# Using Surface Electromyography to Evaluate the Efficacy of Governor Vessel Electroacupuncture in Poststroke Lower Limb Spasticity: Study Protocol for a Randomized Controlled Parallel Trial

**DOI:** 10.1155/2021/5511031

**Published:** 2021-05-24

**Authors:** Jingwen Li, Kaiqi Su, Jinjin Mei, Yiying Wang, Shuai Yin, Yanchao Hu, Wenxue Hao, Xiaodong Feng, Ruiqing Li

**Affiliations:** ^1^Henan University of Chinese Medicine, No. 156 East Jinshui Road, Zhengzhou 450000, China; ^2^Rehabilitation Center, The First Affiliated Hospital of Henan, University of Chinese Medicine, No. 19 Renmin Road, Zhengzhou 450000, China

## Abstract

*Background*. Lower limb spasticity is a common complication after stroke, which seriously affects the quality of life and rehabilitation of patients. There are different treatment methods for poststroke spasticity. It has been found in clinical practice that governor vessel electroacupuncture (GV-EA) can effectively relieve poststroke upper extremity spasticity, but the efficacy of treatment of lower extremity spasticity needs to be further verified. This study aims to design a randomized controlled trial to evaluate the efficacy of GV-EA in the treatment of poststroke lower limb spasticity. *Methods/Design*. This is a randomized, controlled trial. Patients (*N* = 177) will be randomized to receive routine therapeutic drug and rehabilitation treatment plus GV-EA (experimental group) or routine therapeutic drug and rehabilitation treatment plus EA (control group 1) or routine therapeutic drug and rehabilitation treatment (control group 2). All patients will receive 20 sessions of treatment for 4 weeks. The primary outcomes are the RMS value and the Modified Ashworth Scale. Secondary outcomes include the Fugl–Meyer Assessment for Lower Extremity (FMA-LE) and the Modified Barthel Index score. All outcome measures will be evaluated at the beginning and after the intervention (4 weeks). *Discussion*. This trial will observe the clinical effect of GV-EA on lower extremity spasticity after stroke, especially its influence on surface electromyography characteristics, and provide high-quality experimental evidence for the clinical application of GV-EA based on surface electromyography in the treatment of poststroke lower limb spasticity. *Trial Registration*. China Clinical Trials Registry No. ChiCTR1900027969. Registered on 7 December 2019.

## 1. Introduction

Due to the aging population, accelerated urbanization process, and residents' often-unhealthy lifestyles, there is an increased incidence of stroke [1]. According to statistics, 15 million people worldwide die each year from stroke [2]. Spasticity is one of the most common poststroke complications, and it is also an important cause of motor dysfunction. Among them, lower limb spasticity is manifested as muscle atrophy, pain, and joint contracture stiffness [3, 4]. Although lower limb spasticity is not life-threatening, it has become an important public health issue worldwide because of its high recurrence rate and disability, detrimental effects on functional recovery and quality of life, and high health care costs [5]. Epidemiological surveys have shown that the incidence of lower limb spasticity in Western countries after 6 months of stroke patients was about 42.6%, while as high as 65.7% in China [6, 7]. According to statistics, the medical expenses were 4 times higher for stroke patients with spasticity than those of patients without spasticity [8, 9]. Physical therapy, pharmacological therapies, and botulinum toxin seem to be the most common choices for the treatment of lower limb spasticity. However, every conventional approach has its limitations. There is no uniform standard for the treatment parameters and stimulation intensity of physical therapy. Pharmacological therapies have potential side effects, such as drowsiness, nausea, and vomiting. Long-term injection of botulinum toxin may cause serious adverse reactions such as muscle weakness and dyspnea [10]. Therefore, there is a need for a safer, more effective, and less side effect treatment method for poststroke lower limb spasticity.

Acupuncture has been widely applied to treat poststroke spasticity; it is considered by the World Health Organization (WHO) as an alternative and complementary strategy for the treatment and improvement of stroke [11]. Besides, clinical trials and meta-analysis results have demonstrated that acupuncture can improve balance function, reduce spasticity, increase muscle strength, and improve quality of life [12]. It is also more and more popular with clinicians and patients for its unique advantages of significant efficacy, low price, and small side effects. However, the efficacy differences between different acupoints are uncertain. Few trials have involved the comparison of different acupuncture points to verify the efficacy differences between different acupuncture methods. Governor vessel (GV), also known as “the sea of the yang meridians,” is one of the eight extra meridians. GV passes through the kidney, spinal cord, and brain, so it can treat various system diseases closely related to these parts [13]. Our previous study has demonstrated that GV-EA can significantly reduce the root mean square value of the biceps and triceps and the Modified Ashworth Scale score and increase the Fugl–Meyer Assessment for upper extremity motor function score and the Modified Barthel Index score, which can effectively improve the degree of upper limb spasticity, motor function, and ability of daily life [14]. At the same time, studies in modern medicine have shown that GV-EA effectively increases blood flow and partial pressure of oxygen in the brain, thereby accelerating the self-repair ability of the brain tissue. It also has a protective effect on neuronal damage after cerebral ischemia and promotes brain function reorganization [15].

The methods of clinical assessment of limb spasticity mainly include subjective evaluation methods and objective evaluation methods. Among them, the objective evaluation methods include neurophysiological measures and biomechanical measures. The methods mainly rely on certain instruments to determine the severity of spasticity through corresponding measurement indicators, which can make up for the lack of standardization and precision of subjective evaluation scales [16]. Therefore, we selected acupuncture points frequently used clinically and rehabilitation treatment as the control group to design the clinical trial to explore the efficacy of GV-EA in poststroke lower limb spasticity based on sEMG technology, which can provide a strong evidence-based medical basis for further popularizing the application of GV-EA in the treatment of lower limb spasticity in patients with stroke.

## 2. Methods and Analysis

### 2.1. Trial Design

**Figure 1:**
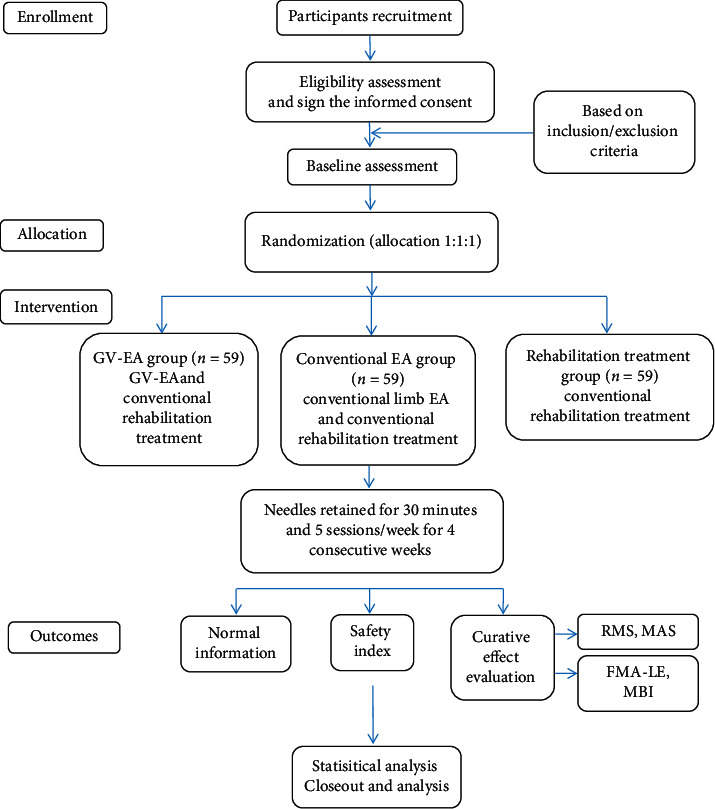
Flowchart of the trial. GV-EA: Governor Vessel electroacupuncture; EA: electroacupuncture; RMS: root mean square; MAS: the Modified Ashworth Scale; FMA-LE: the Fugl–Meyer Assessment for Lower Extremity; MBI: the Modified Barthel Index.

This is a parallel-design and randomized controlled trial. The subjects will be recruited from the Rehabilitation Center of the First Affiliated Hospital of the Henan University of Chinese Medicine. We selected 177 patients who meet the predefined criteria and randomly divided them into 3 groups, with 59 patients in each group: experimental group (GV-EA), control group 1 (conventional EA), and control group 2 (rehabilitation treatment). All patients will receive 20 sessions of treatment that last for 4 weeks and will have sEMG measurements before and after treatment. The efficacy of GV-EA relative to conventional EA and rehabilitation treatment will be analyzed after data collection. The study flow chart is shown in Figure 1. An example template for the content of admission plans, interventions, and evaluations is shown in Table 1. This protocol is guided by the Standard Protocol Items: Recommendations for Interventional Trials (SPIRIT) [17].

### 2.2. Randomization

All included participants will be randomly assigned to three groups at a ratio of 1:1:1: experimental group and control group 1 and control group 2 following the randomization principle, which employs block randomization to generate random-number sequence using SPSS software and an independent statistician who is not involved in treatment and outcome assessment will be responsible for processing the results. Meanwhile, it prepares predetermined sealed opaque envelopes and these envelopes containing the group information strips will be hidden until informed consent is obtained.

### 2.3. Blinding

This is a single-blinded study; because of the nature of acupuncture treatment, none of the acupuncturists involved in this trial can be blinded to the assignments. Patients will be told that they will receive one of the three effective interventions randomized after enrolment. Each patient will know the type of treatment that they accepted, but they will not know the other two types. The intervention of each patient will be performed in a personal space separated to refrain the communication between patients and will be asked to wear an eye patch when they receive treatment. Data managers and statisticians are not clear on the allocation of patient groups. Therapists, data managers, and statisticians are not allowed to communicate with others about the treatment of patients.

### 2.4. Recruitment

Posters, pictures, and videos related to the study will be produced to help participants understand the purpose and program of this study while explaining the advantages and disadvantages of the treatment and relevant safety measures to be taken during the trial. In addition, we will use websites and hospital-based WeChat ads for recruitment. According to the above-mentioned inclusion and exclusion criteria, we will make a preliminary judgment and screening of the possibility of inclusion for each participant and finally determine whether the subject is included based on the results of the relevant examination.

### 2.5. Inclusion and Exclusion Criteria

#### 2.5.1. Inclusion Criteria

Participants meeting the following criteria will be included:

1. Patients who meet the diagnostic criteria of stroke by computed tomography (CT) or magnetic resonance imaging (MRI) (including ischemic and hemorrhagic stroke).
2. Patients with the Modified Ashworth Scale of grade I and above, grade III and below.
3. First onset, subacute stage of stroke (the onset time is 3 weeks to 6 months).
4. Males or females with an age range between 20 and 70 years.
5. Patients with stable vital signs, no cognitive impairment, and cooperate with treatment.
6. Patients who voluntarily agree and sign the informed consent form.

#### 2.5.2. Exclusion Criteria

Participants will be excluded if they meet any of the following criteria:

1. Patients with other serious diseases that may affect treatment outcomes and life-threatening, such as cardiac disease, hepatic disease, pulmonary disease, renal disease, diabetes, osteoporosis, or bleeding tendency.
2. Patients with systemic infection, or severely unstable.
3. Patients who are pregnant, lactating, or preparing for pregnancy.
4. Patients with severe cognition obstacles who cannot cooperate with the treatment.
5. Patients who are participating in other clinical trials.

### 2.6. Interventions

The trial is divided into 3 groups, namely, experimental group (GV-EA group), control group 1 (conventional EA group), and control group 2 (rehabilitation treatment group). These three groups of patients will receive different treatments 5 times a week for 4 weeks in separate compartments. Meanwhile, each patient will receive the same routine therapeutic drug and rehabilitation treatment. In addition to these two treatments, the experimental group also has electroacupuncture on governor vessel acupoints, and the control group 1 has electroacupuncture on conventional acupoints.

#### 2.6.1. Routine Therapeutic Drug Treatment

All patients use blood pressure control, blood lipid adjustment, blood sugar control, and nutritional nerve drug as routine therapeutic drug treatment following the physicians' instruction.

#### 2.6.2. Rehabilitation Treatment

According to the actual situation of all patients, we will follow the theory of neurodevelopment and provide each patient with the same rehabilitation treatment that will include the affected limb position, passive stretching exercises, muscle strength training, proprioceptive neuromuscular facilitation exercises, Bobath therapy exercises, and Brunnstrom therapy exercises.

#### 2.6.3. Acupuncture Interventions

In China, stroke patients are familiar with acupuncture treatment. Thus, it is easy for patients to determine whether fake acupuncture is used. And because an important purpose of this trial is to make up for the lack of comparison of the efficacy differences between different acupuncture acupoints in the current experiment, placebo acupuncture was not selected. Therefore, we decided to perform clinical conventional electroacupuncture as the control group rather than sham acupuncture and placebo acupuncture.

The interventions will be performed by acupuncturists with more than 3 years of experience in rich clinical acupuncture practice. The acupuncture treatment group used sterilized stainless steel disposable acupuncture needles (0.3∗30 mm). After the needle is inserted to a certain depth of points, it will be lifted and thrust or twirled and rotated for Deqi sensation. After feeling of “Deqi”(the sensations including soreness, numbness, distention, or heaviness), it was connected to the electroacupuncture device (G6805-2A, Shanghai Huayi Medical Instrument Co., Ltd., China, dense waves 2–5 times/second, frequency 2.5 Hz), once a day, every 30 minutes, 5 times a week, for 4 weeks.

#### 2.6.4. Experimental Group

In addition to the two routine treatments, acupuncture will also be performed at Dazhui (GV14), Shendao (GV11), Jinsuo (GV8), Mingmen (GV4), and Yaoyangguan (GV3) for patients in the experimental group. The specific operation method is as follows: generally take the prone position, punctured obliquely to a depth of 0.5–1.0 cm. After the needle is inserted, manipulations will be applied for the “Deqi” sensation. The locations are specifically presented in Table 2.

#### 2.6.5. Control Group 1

The acupuncture will also be performed at the hemiplegic side Blood sea (SP10), Zusanli (ST36), Yinlingquan (SP9), Yanglingquan (GB34), and Sanyinjiao (SP6) for patients in control group 1. The selected acupoints in this group were high-frequency acupoints based on data mining, the clinical experience of famous acupuncturists of the past, and theoretical research of Chinese medicine for acupuncture treatment of poststroke spasticity [18]. Specific operation: generally take the supine position, perpendicular insertion 1.0–1.5 cm. After “Deqi”, the electrode will be connected to the needle.

#### 2.6.6. Control Group 2

Control group 2 only received the two routine treatments.

### 2.7. Sample Size

The purpose of this study is to evaluate the efficacy of GV-EA in the treatment of poststroke lower limb spasticity. Based on previous studies and clinical experience, the effectiveness rate of conventional EA and rehabilitation treatment is about 75% and 60%, and it is expected that electroacupuncture of GV acupoint will reach 90% [19]. Type 1 error is assumed at 0.05, type 2 error is assumed at 0.1, and a standard deviation is 10. PASS15.0 software (NCSS Statistical Software, Kaysville, UT, USA) has been used to determine the sample size, and the minimum sample size is 53 subjects for each group. Considering a dropout rate of 10%, a total of 177 subjects will be required with 1:1:1 allocation to each group (59 participants per group) for this study.

### 2.8. Outcome Assessments

The observation indexes of result measurement include three parts: baseline, safety indicators (general physical examination, routine blood tests, routine urine tests, routine stool tests, electrocardiograms, and liver and kidney function tests), and clinical efficacy observation indexes. All evaluations will be performed before and after treatment.

### 2.9. Primary Outcomes

The main result is surface electromyography (sEMG) measurement and the Modified Ashworth Scale (MAS).

### 2.10. Surface Electromyography (sEMG) Measurement

The sEMG, also known as dynamic electromyography, is a widely used technology in rehabilitation research and provides quantifiable information on the myoelectric output of a muscle [20]. It is a noninvasive inspection method for recording bioelectric signals during muscle activity through surface electrodes and is an essential test tool for studying the functional status of the neuromuscular system. At the same time, it provides a scientific basis for clinical treatment. Commonly used evaluation indicators for sEMG include frequency and time-domain indicators, among which time-domain indicators mainly include the root mean square (RMS). Among them, the RMS value reflects the change of the amplitude of the sEMG signal voltage in the time dimension and variation of relevant muscle strength [21]. Its change mainly reflects the number of motor units activated during muscle activity, the type of motor units participating in the activity, and the degree of synchronization. The sEMG can also distinguish between spasticity and contracture. When the joint resistance measured by passive stretching increases and the antagonistic myoelectric signal is not significantly different from the resting state, it is a contracture, and the electromyographic signal is significantly increased, which is spasticity.

Before and after the treatment, the sEMG signal system (BioNeuro Infiniti, Thought Technology Ltd., Canada) was used to collect the electromyographic signal values of the quadriceps, hamstrings, calf triceps, and tibialis anterior muscles of the hemiplegic lower limbs. With the patient in the sitting position, a 75% alcohol cotton swab will be used to clean the skin on the measured muscle surface to remove oil on the skin surface, reduce electrical resistance, and increase the electrical conductivity between the surface electrode and the skin. After cleaning, according to the anatomical position of the muscle and the direction of the muscle fiber, the disposable surface electrode sheets are, respectively, pasted on the muscle belly of the measured muscle. Select the program measurement mode, passively stretch the tested muscles, passively stretch 3 times, relax 3 times, 5 s each time, take RMS value as the detection index, and calculate cocontraction ratios (CCR); the specific calculation formula is

CCR = antagonist muscle RMS/(active muscle RMS + antagonist muscle RMS)

## 3. The Modified Ashworth Scale (MAS) [22]

The MAS is the most widely used spasticity assessment and has the advantages of simplicity, time savings, and convenient operation, which includes 6 levels. To facilitate data recording and analysis and statistical analysis, grades 0, I, I+, II, III, and IV are converted into 0, 1, 2, 3, 4, and 5 points, respectively. The higher the MAS score is, the more serious the spasticity is considered as shown in Table 3.

### 3.1. Secondary Outcomes

The Fugl–Meyer Assessment for Lower Extremity (FMA-LE) and the Modified Barthel Index score (MBI) were the secondary observation outcome indicators.

## 4. The Fugl–Meyer Assessment for Lower Extremity (FMA-LE)

The FMA-LE is a widely recognized and valid clinical measure of poststroke motor impairment severity assessment. The lower extremity has a maximum score of 34 points divided into motor, balance, sensory, range of motion, and joint pain and a higher score indicates better lower limb movement function [23, 24]. The FMA-LE is the most common scale for evaluating lower limb motor function after stroke.

### 4.1. The Modified Barthel Index Score (MBI)

The MBI is mainly used to assess the patient's daily living ability, including control urination, eating, grooming, toileting, transfer, walking, dressing, bathing, and going up and down stairs. The total score is 100 points. A higher score indicates the worst daily living ability. The MBI classification can be divided into without dependence (100 points), mild dependence (61∼99 points), moderate dependence (41∼60 points), severe dependence (1∼40 points), and completely dependent (0 points) [25].

### 4.2. Safety Evaluation and Adverse Events

Any adverse events and how they are dealt with will be recorded throughout the treatment process. Adverse events related to acupuncture treatment included severe pain, fainting, bleeding, or other any discomfort. During the intervention period, the researchers will pay close attention to the patient's condition. If any discomfort occurs to the patient, the intervention will be stopped immediately and the patient's condition will be dealt with accordingly. Any medical occurrence detailed information will be reported in detail in case report forms (CRFs). The primary researchers will review all adverse events periodically, and the Ethics Committee will have access to the interim results.

### 4.3. Data Collection and Management

The data of all patients will be recorded on the CRFs, which include observation time points, outcome measures, adverse events, safety evaluations. Two independent researchers will input the data into the Excel spreadsheet, and each patient's personal privacy information will be protected. Data will be safely kept by the data researchers of our team and monitored by the Ethics Committee of the First Affiliated Hospital of the Henan University of Chinese Medicine each year as shown in Table 1.

### 4.4. Statistical Analysis

Statistical analysis will be performed using SPSS 25.0 software for statistical analysis. To measure baseline, a chi-square test or analysis of variance (ANOVA) is used to compare demographic data and other basic data at baseline. Continuous variables are expressed in terms of mean ± standard deviation when following a normal distribution. If not, the data will be shown by medians and interquartile ranges. Frequencies and percentages will be used to count the data. The paired *t*-test will be used to compare the differences between the groups before and after treatment, including the RMS value, FMA-LE, and MBI score. The comparison among the three groups before and after treatment will be analyzed by variance (ANOVA). For the MAS, the Kruskal–Wallis test will be used. The test level is set to 0.05, and *P* < 0.05 means the difference is statistically significant.

### 4.5. Quality Control

To reduce the potential bias and ensure the quality of this trial, all acupuncturists and assessors will be required to receive standard training prior to the beginning of the trial. The content of the training course is provided by the protocol, including recruitment, interventions, and assessment process. Whether the data is complete and accurate will be monitor by a qualified clinical trial expert from the Clinical Research Center of the First Affiliated Hospital of the Henan University of Chinese Medicine. All clinical trial experts are independent of the sponsor. In addition, a quality control team will be established to supervise whether the experimental procedure meets the standard guideline. Statistical analysis will be performed when the total number of samples collected reaches 89 cases. The primary investigator will obtain these interim results and will decide whether to continue the trial. We will stop the trial if the efficacy of the patients in the experimental group is much lower than the other two groups in the outcomes of the interim data [26].

## 5. Discussion

Lower limb spasticity will severely limit the patient's trunk antigravity activity function and daily and social life ability recovery and even induce severe complications such as pneumonia and deep vein thrombosis [27]. It is of great significance to find effective treatment measures to reduce lower limb spasticity after stroke. This study is designed to evaluate the efficacy and safety of GV-EA in the treatment of poststroke lower limb spasticity.

### 5.1. Acupuncture Points Selection

The GV walks in the middle of the back and intersects with the three yang meridians of hand and foot and the Yang Link Vessel many times. Due to the connection between the GV and the yang meridians, it is also called “the sea of the yang meridians” [28]. Unblock Yang takes the regulation of Du as the first, so acupoints on the GV are selected. In this trial, the selected acupoints GV14, GV11, GV8, GV4, and GV3 are all the GV points. Among them, GV14 is the intersection of the GV and the three yang meridians of hand and foot, that is, “confluence of the yang”. GV8 acupoints mainly treat convulsions, strong spine, limbs, and tendons and have a good effect on spastic hemiplegia caused by stroke [29, 30]. EA at the above-mentioned acupoints can effectively promote the circulation of yang qi in the GV to the whole body. Blood flows with qi, and qi and blood flow to the end of the limbs so that the muscles and veins of the limbs can be nourished by qi and blood, thereby relieving limb spasticity.

### 5.2. Outcome Measurements Selection: Surface Electromyography (sEMG)

The sEMG evaluation is an objective method to obtain the electromyographic signal during muscle activity, which is achieved by placing surface electrodes on the muscle layer [31]. It can perform quantitative and qualitative analysis on the function of muscles and can study multiple muscles on the body at the same time. It has the advantages of noninvasiveness, safety and reliability, convenient operation, and objective quantification [32]. The evaluation of nerve and muscle function and rehabilitation effects of stroke patients has gained more and more attention. The muscle strength classification scale and muscle spasticity detection methods that are still in use until now are subject to great subjectivity; the detection results are difficult to accurately quantify and qualify so that subjective evaluation methods are questionable and limited in clinical applications [33]. And through the analysis of sEMG signal, it can reflect the level of motor unit recruitment and synchronization and can study the functional status and control mechanism of the nerve and muscle of stroke patients with hemiplegia to evaluate the rehabilitation effect and guide that rehabilitation process has become an important tool in the field of rehabilitation [34, 35].

One important limitation of our study is that as it is an acupuncture trial, it is impossible to execute double-blind procedures. To reduce this kind of bias, each group of patients will be treated in a different room, and each group of patients will be prohibited from talking. In addition, all the acupuncturists participating in this study will conduct unified training and strictly regulate the acupuncture operation to maximize the elimination bias. In addition to the required scales, sEMG is also used in the evaluation to reduce the interference of subjective factors. We expect that the results of this trial will provide evidence on the efficacy and safety of GV-EA in the treatment of poststroke lower limb spasticity. This will provide a practical and effective treatment method for the clinic, which has very important social significance.

## Figures and Tables

**Table 1:** Qualification screening and informed consent will be completed before the assignment. After allocation, each patient will be treated within 4 weeks. The clinical outcome will be evaluated twice: after allocation and after treatment. Adverse events will be recorded on the case report form at any time during treatment.

Timepoint	Enrollment	Allocation	Treatment period			
	−1 week	0 weeks	1 week	2 weeks	3 weeks	4 weeks
*Enrollment*						
Eligibility screen	X					
Baseline	X					
Informed consent	X					
Medical history	X					
Merger disease	X					
Allocation		X				

*Interventions*						
Group A			X	X	X	X
Group B			X	X	X	X
Group C			X	X	X	X

*Assessments*						
RMS		X				X
MAS		X				X
FMA		X				X
MBI		X				X
Safety evaluation		X				X
Needle sensation			X	X	X	X
Adverse events			X	X	X	X

**Table 2:** Location of acupoints for treating poststroke lower limb spasticity.

Acupoints location	
Dazhui (GV14)	In the depression inferior to the spinous process of the seventh cervical vertebra (C7), on the posterior median line
Shendao (GV11)	In the depression inferior to the spinous process of the fifth thoracic vertebra (T5), on the posterior median line
Jinsuo (GV8)	In the depression inferior to the spinous process of the ninth thoracic vertebra (T9), on the posterior median line
Mingmen (GV4)	In the depression inferior to the spinous process of the second lumbar vertebra (L2), on the posterior median line
Yaoyangguan (GV3)	In the depression inferior to the spinous process of the fourth lumbar vertebra (L4), on the posterior median line

**Table 3:** The MAS specific scoring rules.

Modified Ashworth Scale	
1	No increase in muscle tone
1	Slight increase in muscle tone, manifested by a catch or by minimal resistance at the end of the range of motion (ROM) when the affected part(s) is (are) moved in flexion or extension
1+	Slight increase in muscle tone, manifested by a catch, followed by minimal resistance throughout the remainder (less than half) of the ROM
2	More marked increase in muscle tone through most of the ROM, but affected part(s) easily moved
3	Considerable increase in muscle tone, passive movement difficult
4	Affected part(s) rigid in flexion or extension

## Data Availability

No data were used to support this study.

## Abbreviations

GV-EA: Governor vessel electroacupuncture
EA: Electroacupuncture
sEMG: Surface electromyography
SPIRIT: Standard Protocol Items: Recommendations for Interventional Trials
CT: Computed tomography
MRI: Magnetic resonance imaging
PASS: Power analysis and sample size
SPSS: Statistical Package for the Social Sciences
RMS: Root mean square
MAS: Modified Ashworth Scale
FMA-LE: Fugl–Meyer Assessment for Lower Extremity
MBI: Modified Barthel Index
CRFs: Case report forms.

## Supplementary Materials

Supplementary Materials Additional file 1: Completed Standard Protocol Items: Recommendation for Interventional Trials (SPIRIT) 2013 Checklist: items addressed in this clinical trial protocol. Additional file 2: STRICTA 2010 checklist of information to include when reporting interventions in a clinical trial of acupuncture (expansion of Item 5 from CONSORT 2010 checklist). Additional file 3: Informed consent form.

## References

[1] Virani S. S., Alonso A., Benjamin E. J. (2020). Heart disease and stroke statistics-2020 update: a report from the American heart association. *Circulation*.

[2] Johnson C. O., Nguyen M., Roth G. A. (2019). Global, regional, and national burden of stroke, 1990-2016: a systematic analysis for the Global Burden of Disease Study 2016. *Lancet Neurol*.

[3] Gorst T., Rogers A., Morrison S. C. (2019). The prevalence, distribution, and functional importance of lower limb somatosensory impairments in chronic stroke survivors: a cross sectional observational study. *Disability and Rehabilitation*.

[4] Jung T. M., Kim A. R., Lee Y., Kim D.-H., Kim D. Y. (2016). Precise muscle selection using dynamic polyelectromyography for treatment of post-stroke dystonia: a case report. *Annals of Rehabilitation Medicine*.

[5] Ghannadi S., Shariat A., Ansari N. N. (2020). The effect of dry needling on lower limb dysfunction in poststroke survivors. *Journal of Stroke and Cerebrovascular Diseases*.

[6] Urban P. P., Wolf T., Uebele M. (2010). Occurence and clinical predictors of spasticity after ischemic stroke. *Stroke*.

[7] Cui L. H., Shan L., Yang Y. Q. (2014). Incidence of spasticity after first stroke within 6 Months. *Chinese Journal of Rehabilitation Theory and Practice*.

[8] Angerova Y., Marsalek P., Chmelova I. (2020). Cost and cost-effectiveness of early inpatient rehabilitation after stroke varies with initial disability: the Czech Republic perspective. *International Journal of Rehabilitation Research*.

[9] Ganapathy V., Graham G. D., DiBonaventura M. D., Gillard P. J., Goren A., Zorowitz R. D. (2015). Caregiver burden, productivity loss, and indirect costs associated with caring for patients with poststroke spasticity. *Clinical Interventions in Aging*.

[10] Sunnerhagen K. S., Olver J., Francisco G. E. (2013). Assessing and treating functional impairment in poststroke spasticity. *Neurology*.

[11] Chavez L., Huang S.-S., MacDonald I., Lin J.-G., Lee Y.-C., Chen Y.-H. (2017). Mechanisms of acupuncture therapy in ischemic stroke rehabilitation: a literature review of basic studies. *International Journal of Molecular Sciences*.

[12] Cai Y., Zhang C. S., Liu S. (2017). Electroacupuncture for poststroke spasticity: a systematic review and meta-analysis. *Archives of Physical Medicine and Rehabilitation*.

[13] Wang Y. P., Zhang W. B., Li H. Y. (2020). Analysis on the route of conception vessel and governor vessel in Huangdi neijing (The Yellow Emperor’s Inner Classic). *Chinese Acupuncture & Moxibustion*.

[14] Li R. Q., Liu C. M., Xi J. M. (2019). Effects of electro-acupuncture at Du meridian in stroke patients with upper-extremity spasticity and its character of sEMG[J]. *Chinese Journal of Rehabilitation Medicine*.

[15] Pan J., Chen W. S., Chen C. (2017). Effects of electric-acupuncture at du-meridian on MCAO rats infarct volume and NGF in brain tissue. *Chinese Archives of Traditional Chinese Medicine*.

[16] Chan A.-W., Tetzlaff J. M., Gotzsche P. C. (2013). SPIRIT 2013 explanation and elaboration: guidance for protocols of clinical trials. *BMJ*.

[17] Thibaut A., Chatelle C., Ziegler E., Bruno M.-A., Laureys S., Gosseries O. (2013). Spasticity after stroke: physiology, assessment and treatment. *Brain Injury*.

[18] Zhang Y., Zhang Z., Wang X. (2018). Exploration on the characteristics of meridian points in the treatment of Spasticity paralysis after stroke with acupuncture and moxibustion based on the data mining technology. *Inner Mongolia Journal of Traditional Chinese Medicine*.

[19] Xu L., Wang M., Li F., Yang J. (2017). Acupuncture combined with rehabilitation training for the limb spasm after stroke. *Zhongguo Zhen Jiu*.

[20] Pilkar R., Momeni K., Ramanujam A., Ravi M., Garbarini E., Forrest G. F. (2020). Use of surface EMG in clinical rehabilitation of individuals with SCI: barriers and future considerations. *Frontiers in Neurology*.

[21] Ma X., Liu Y., Song Q., Wang C. (2020). Continuous estimation of knee joint angle based on surface electromyography using a long short-term memory neural network and time-advanced feature. *Sensors*.

[22] Pandyan A. D., Johnson G. R., Price C. I. M., Curless R. H., Barnes M. P., Rodgers H. (1999). A review of the properties and limitations of the Ashworth and modified Ashworth Scales as measures of spasticity. *Clinical Rehabilitation*.

[23] Hernández E. D., Forero S. M., Galeano C. P., Barbosa N. E., Sunnerhagen K. S., Alt Murphy M. (2020). Intra- and interrater reliability of fugl-meyer assessment of lower extremity early after stroke. *Brazilian Journal of Physical Therapy*.

[24] Sullivan K. J., Tilson J. K., Cen S. Y. (2011). Fugl-meyer assessment of sensorimotor function after stroke. *Stroke*.

[25] Granger C. V., Hamilton B. B., Gresham G. E. (1988). The stroke rehabilitation outcome study--Part I: general description. *Archives of Physical Medicine and Rehabilitation*.

[26] Su KQ., Liu ST., Li JY. (2021). Effects of different acupuncture treatment methods on post-stroke cognitive impairment: study protocol for a multicenter randomized controlled trial. *Trials*.

[27] Ashford S., Williams H., Nair A., Orridge S., Turner-Stokes L. (2019). Categorisation of goals set using Goal Attainment Scaling for treatment of leg spasticity: a multicentre analysis. *Disability and Rehabilitation*.

[28] Li J., He S. H., Tang M. L. (2019). Talking about the relationship between Du channel’s “yangmai sea” theory and nerve distribution and related diseases from the three yang meridians of hand and foot. *Guanxi Medical Journal*.

[29] Xin Y. X., Chen C. H., Shi H. M. (2018). General situation of clinical application of Du channel therapy. *Chinese Journal of Traditional Medical Science and Technology*.

[30] Ma H. F., Li R. (1999). Discussion on the rule of indications of Du channel meridian points. *Journal of Beijing University ofTCM*.

[31] Tang X., Zhang X., Gao X., Chen X., Zhou P. (2018). A novel interpretation of sample entropy in surface electromyographic examination of complex neuromuscular alternations in subacute and chronic stroke. *IEEE Transactions on Neural Systems and Rehabilitation Engineering*.

[32] McManus L., De Vito G., Lowery M. M. (2020). Analysis and biophysics of surface EMG for physiotherapists and kinesiologists: toward a common language with rehabilitation engineers. *Frontiers in Neurology*.

[33] Hong M. J., Park J. B., Lee Y. J. (2018). Quantitative evaluation of post-stroke spasticity using neurophysiological and radiological tools: a pilot study. *Annals of Rehabilitation Medicine*.

[34] Hogrel J.-Y. (2005). Clinical applications of surface electromyography in neuromuscular disorders. *Neurophysiologie Clinique/Clinical Neurophysiology*.

[35] Klein C. S., Li S., Hu X., Li X. (2018). Editorial: electromyography (EMG) techniques for the assessment and rehabilitation of motor impairment following stroke. *Frontiers in Neurology*.

