# Supplementary material for: Using Surface Electromyography to Evaluate the Efficacy of Governor Vessel Electroacupuncture in Poststroke Lower Limb Spasticity: Study Protocol for a Randomized Controlled Parallel Trial
Source: Evid Based Complement Alternat Med. 2021 May 24;2021:5511031. doi: 10.1155/2021/5511031 (PMC8166481; doi:10.1155/2021/5511031)
Supplement: Supplementary Materials — Additional file 1: Completed Standard Protocol Items: Recommendation for Interventional Trials (SPIRIT) 2013 Checklist: items addressed in this clinical trial protocol. Additional file 2: STRICTA 2010 checklist of information to include when reporting interventions in a clinical trial of acupuncture (expansion of Item 5 from CONSORT 2010 checklist). Additional file 3: Informed consent form. [file 5511031.f1.zip › 5511031.f1/Additional file 2 STRICTA 2010.docx]

**Additional file 2: Checklist for items in Standards for Reporting Interventions in Clinical Trials of Acupuncture(** **STRICTA 2010 )**

| **Item** | **Detail** | **Page number** |
| --- | --- | --- |
| **1. Acupuncture rationale** | 1a) Style of acupuncture (e.g. Traditional Chinese Medicine, Japanese, Korean, Western medical, Five Element, ear acupuncture, etc) | 3-4 |
|  | 1b) Reasoning for treatment provided, based on historical context, literature sources, and/or consensus methods, with references where appropriate | 3-4 |
|  | 1c) Extent to which treatment was varied | 3-4 |
| **2. Details of needling** | 2a) Number of needle insertions per subject per session (mean and range where relevant) | 10 |
|  | 2b) Names (or location if no standard name) of points used (uni/bilateral) | 10-12, Table 2 |
|  | 2c) Depth of insertion, based on a specified unit of measurement, or on a particular tissue level | 10-12 |
|  | 2d) Response sought (e.g. *de qi* or muscle twitch response) | 10 |
|  | 2e) Needle stimulation (e.g. manual, electrical) | 10 |
|  | 2f) Needle retention time | 10 |
|  | 2g) Needle type (diameter, length, and manufacturer or material) | 10 |
| **3. Treatment regimen** | 3a) Number of treatment sessions | 10 |
|  | 3b) Frequency and duration of treatment sessions | 10 |
| **4. Other components of treatment** | 4a) Details of other interventions administered to the acupuncture group (e.g. moxibustion, cupping, herbs, exercises, lifestyle advice) | 9-12 |
|  | 4b) Setting and context of treatment, including instructions to practitioners, and information and explanations to patients | 8,10 |
| **5. Practitioner background** | 5) Description of participating acupuncturists (qualification or professional affiliation, years in acupuncture practice, other relevant experience) | 10,17 |
| **6. Control or comparator interventions** | 6a) Rationale for the control or comparator in the context of the research question, with sources that justify this choice | 3,4 |
|  | 6b) Precise description of the control or comparator. If sham acupuncture or any other type of acupuncture-like control is used, provide details as for Items 1 to 3 above. | 9-12 |
