# Supplementary material for: Using Surface Electromyography to Evaluate the Efficacy of Governor Vessel Electroacupuncture in Poststroke Lower Limb Spasticity: Study Protocol for a Randomized Controlled Parallel Trial
Source: Evid Based Complement Alternat Med. 2021 May 24;2021:5511031. doi: 10.1155/2021/5511031 (PMC8166481; doi:10.1155/2021/5511031)
Supplement: Supplementary Materials — Additional file 1: Completed Standard Protocol Items: Recommendation for Interventional Trials (SPIRIT) 2013 Checklist: items addressed in this clinical trial protocol. Additional file 2: STRICTA 2010 checklist of information to include when reporting interventions in a clinical trial of acupuncture (expansion of Item 5 from CONSORT 2010 checklist). Additional file 3: Informed consent form. [file 5511031.f1.zip › 5511031.f1/Additional file 3 Consent Form.docx]

**Consent Form**

**Research name**: Using surface electromyography to evaluate the efficacy of governor vessel electroacupuncture in post-stroke lower limb spasticity: study protocol for a randomized controlled parallel trial

**Research number**: grant number 2019HL-103-01

**Research institute**: Henan University of Chinese Medicine; Rehabilitation Center of the First Affiliated Hospital of Henan University of Chinese Medicine

You will be invited to participate in a clinical study. This informed consent gives you some information to help you decide whether to participate in this clinical study or not. Please read it carefully. If you have any questions, please ask the researchers responsible for the study.

Your participation in this study is voluntary. This study has been reviewed by the ethics review committee of the research institute. If you have questions related to the subjects' rights and interests, please contact the Ethics Committee of the First Affiliated Hospital of Henan University of Chinese Medicine. Contact number: 0371-66285929.

Research purpose: This proposed randomized controlled trial (RCT), intends to evaluate the efficacy of GV-EA in post-stroke lower limb spasticity based on sEMG technology, which can provide a strong evidence-based medical basis for further popularizing the application of GV-EA in the treatment of lower limb spasticity in patients with stroke.

Research process: If you agree to participate in this study, we will number each subject and create a medical record file. You will receive 20 sessions of treatment for 4 weeks.

Risk and Privacy issue: Risks associated with this study are minimal. If you decide to participate in this study, your personal data in and during the study are confidential. You may choose not to participate in this study, or at any time inform the researcher to request withdrawal from the study. Your data will not be included in the study results, and any medical treatment and benefits will not be affected.

***If you'd like to take part in the study, You can sign this informed consent:***

Patient's name：________________________

Signature of patient：____________________

Date：

Researcher's name:____________________

Signature of researcher:____________________

Date:
